# Yellow Fever Virus Genotyping Tool and Investigation of Suspected Adverse Events Following Yellow Fever Vaccination

**DOI:** 10.3390/vaccines7040206

**Published:** 2019-12-04

**Authors:** Izabela Maurício de Rezende, Pedro Augusto Alves, Matheus Soares Arruda, Andreza Parreiras Gonçalves, Gabriela Fernanda Garcia Oliveira, Leonardo Soares Pereira, Maria Rita Teixeira Dutra, Ana Carolina Campi-Azevedo, Valéria Valim, Renata Tourinho, Jaquelline Germano de Oliveira, Carlos Eduardo Calzavara, Rodrigo Fabiano do Carmo Said, Erna Geessien Kroon, Olindo Assis Martins-Filho, Andrea Teixeira-Carvalho, Betânia Paiva Drumond

**Affiliations:** 1 Departament of Microbiology, Universidade Federal de Minas Gerais, 31270-901 Belo Horizonte, Minas Gerais, Brazil; izabelamauriciorezende@gmail.com (I.M.d.R.); matheusmtsa@hotmail.com (M.S.A.); gabrielafernandag@gmail.com (G.F.G.O.); leosoaresgallo@yahoo.com.br (L.S.P.); ernagkroon@gmail.com (E.G.K.); 2 Instituto René Rachou/Fundação Oswaldo Cruz, 30190-002 Belo Horizonte, Minas Gerais, Brazil; pedroaugustoalves@yahoo.com.br (P.A.A.); andreza.parreiras@hotmail.com (A.P.G.); campiazevedo@gmail.com (A.C.C.-A.); jaquelline.oliveira@fiocruz.br (J.G.d.O.); calzavara@gmail.com (C.E.C.); oamfilho@gmail.com (O.A.M.-F.); atcteixeira@gmail.com (A.T.-C.); 3 Hospital Eduardo de Menezes, Fundação Hospitalar do Estado de Minas Gerais, 30622-020 Belo Horizonte, Minas Gerais, Brazil; mariaritatdutra@gmail.com; 4 Hospital Universitário Cassiano Antônio Moraes - Empresa Brasileira de Serviços Hospitalares, Universidade Federal do Espírito Santo, 29075-910 Vitória, Espírito Santo, Brazil; val.valim@gmail.com; 5 Biomanguinhos/Fundação Oswaldo Cruz, 21040-900 Rio de Janeiro, Rio de Janeiro, Brazil; renata.tourinho@bio.fiocruz.br; 6 Secretaria de Estado de Saúde de Minas Gerais, 31630-900 Belo Horizonte, Minas Gerais, Brazil; rodrigo.said@saude.gov.br; 7 Departamento de Vigilância das Doenças Transmissíveis, Secretaria de Vigilância em Saúde, Ministério da Saúde, 70058-900 Brasília, Distrito Federal, Brazil

**Keywords:** yellow fever virus, YFV-17DD, adverse event, arbovirus, yellow fever, vaccination, immunisation, vaccine, genotyping, wild-type virus

## Abstract

The yellow fever (YF) vaccine consists of an attenuated virus, and despite its relative safety, some adverse events following YF vaccination have been described. At the end of 2016, Brazil experienced the most massive sylvatic yellow fever outbreak over the last 70 years and an intense campaign of YF vaccination occurred in Minas Gerais state in Southeast Brazil from 2016 to 2018. The present study aimed to develop a genotyping tool and investigate 21 cases of suspected adverse events following YF vaccination. Initial in silico analyses were performed using partial NS5 nucleotide sequences to verify the discriminatory potential between wild-type and vaccine viruses. Samples from patients were screened for the presence of the YFV RNA, using 5′UTR as the target, and then used for amplification of partial NS5 gene amplification, sequencing, and phylogenetic analysis. Genotyping indicated that 17 suspected cases were infected by the wild-type yellow fever virus, but four cases remained inconclusive. The genotyping tool was efficient in distinguishing the vaccine from wild-type virus, and it has the potential to be used for the differentiation of all yellow fever virus genotypes.

## 1. Introduction

Yellow fever virus (YFV) (Riboviria, family *Flaviviridae*, genus *Flavivirus*) [1] is the causative agent of yellow fever (YF), widely distributed in the tropical and subtropical regions of South America and Africa. In Brazil, YFV is maintained in a sylvatic cycle involving mosquitoes of the genera *Haemogogus*, *Sabethes,* vertebrate hosts as non-human primates (NHP) [2] and humans, sporadically [3]. Although an effective vaccine against YF has been in existence since 1937, the disease is responsible for approximately 200,000 cases and 29,000 to 60,000 deaths annually [2].

The original YFV-17D vaccine is a live-attenuated vaccine that is well-tolerated and considered safe worldwide. The YFV-17D strain is derived from the Asibi strain [4] and served as the basis for the vaccine strains, YFV-17D-204 and YFV-17DD, still in use worldwide. YFV 17D-204 and 17-DD share 99.9% of nucleotide sequence similarity. Analysis of deduced polyprotein sequence of YFV-17DD indicated 20 amino acid substitutions compared to the original Asibi strain. Due to those amino acid differences, YFV vaccine strains are not transmitted by mosquitoes [5,6].

Some reports of adverse events following YF vaccination have been described, being typically mild, including headache, myalgia, low-grade fever, and discomfort at the injection site. Severe adverse events following YF vaccination are rare and can be classified as (i) YF vaccine-associated viscerotropic disease; (ii) YF vaccine-associated neurological diseases, and (iii) hypersensitive reactions [6,7,8]. The viscerotropic adverse event is a severe acute illness with a short incubation period (2–5 days), resembling a natural infection and the vaccinees may present haemorrhage, hepatic insufficiency, hypotension, myocarditis, and renal insufficiency, among others. The predominant type of YF vaccine-associated neurological disease is acute meningoencephalitis. The median onset of clinical signs is 11 days, but the beginning of symptoms can occur up to 30 days following the vaccination [6,9]. In Brazil, from 2007 to 2012, the occurrence of adverse events was estimated as 0.42 events per 100,000 inhabitants [10].

For lifelong protection against YF, for children older than nine months to adults up to 59 years, a single dose of YF vaccine has been recommended [5,11]. YF vaccination stimulates the activation of cellular and humoral immune responses in 99% of vaccinees within 30 days of vaccination [12]. However, at least three studies in Brazil have demonstrated a significant decrease or even a complete absence of neutralising antibody titers, effector memory CD4+ and CD8+ T-cells, and classical memory B-cells ten years after the primary vaccination. These studies altogether demonstrate a fragility of memory responses and reinforce the need for one booster dose ten years after the first YFV-17DD dose, especially for people living in YF risk areas [13,14,15].

Usually, during mass vaccination campaigns, an increase in the number of cases with adverse events following vaccination can be observed [7,11], attributable mainly to a large number of vaccinated people [7]. In YF endemic regions, it is essential to discriminate between serious adverse events and wild-type YFV infection [7,9,11]. YFV genotyping approaches have been proposed using RT-qPCR, for distinguishing South American genotypes from the YF vaccine strains [16] or using RT-qPCR followed by deep sequencing [17]. All vaccinees reporting generalized febrile or neurological illness, headache, body pain, nausea, vomiting, jaundice, bleeding, and others flu-like unspecific symptoms up to 30 days following vaccination should be notified and suspected adverse events investigated [7,9].

At the end of 2016, Brazil experienced the largest sylvatic YF outbreak in 70 years [11,18]. From December 2016 up to June 2019, 2240 human cases and 760 deaths were confirmed in the country [19,20,21], with 1002 cases (44.73%) and 340 deaths reported in Minas Gerais state [11,22]. From 2016 to 2018, an intense campaign of YF vaccination occurred in Minas Gerais, with more than 7.1 million doses applied [23]. During the YF outbreaks in Minas Gerais, the vaccinees presenting the above-mentioned symptoms up to 30 days of vaccination were considered suspected cases of adverse events [23]. The present study aimed to optimize an accessible YFV genotyping tool and investigate suspected cases of adverse events following YFV-17DD vaccination during the recent YF outbreaks (2017–2018) in Minas Gerais.

## 2. Methods

### 2.1. Clinical Samples

During the past YF outbreak, we received sera from suspected cases of adverse events up to 30 days after YF vaccination. The patients looked for medical care at different times after the onset of disease; in that way, the sera were obtained from different moments regarding the infection course and YF vaccination. The earliest available sample of each patient was used to perform viral genotyping. In addition, sera obtained from health primary vaccinees with confirmed YFV-17DD viremia (*n* = 25) and from patients with confirmed wild-type YFV infection (*n* = 4) were used as positive controls.

This project was approved by the Ethics Committee for studies with human subjects at Instituto René Rachou—IRR/FIOCRUZ-MG (number CAAE 72569317.2.0000.5091, and CAAE: 65910317.0000.5071; Rede Brasileira de Ensaios Clínicos-REBEC: U1111-1217-6672).

### 2.2. Yellow Fever Virus Molecular Screening 

**Figure 1:**
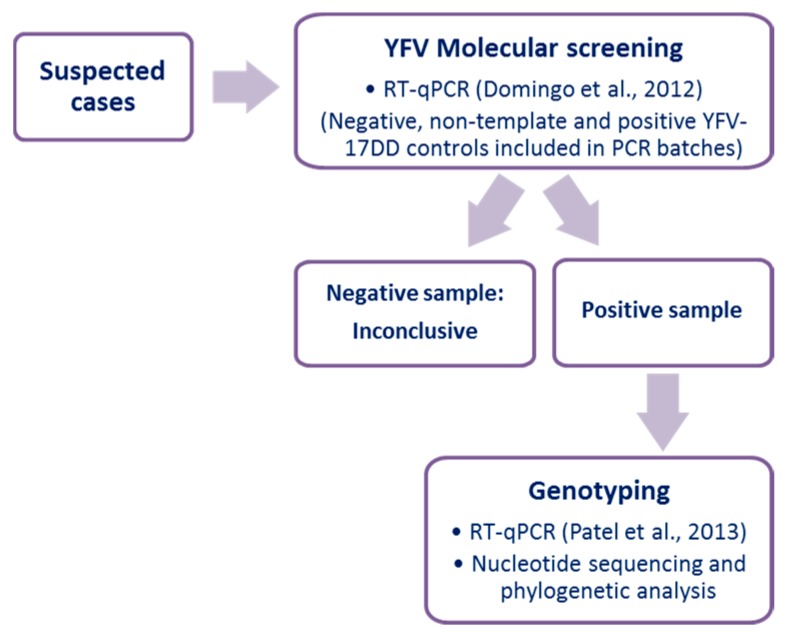
Flowchart for analysis of suspected cases of adverse event following yellow fever vaccination.

Since the samples were obtained at different moments of the disease course, the strategy used here included initial molecular testing for the presence of YFV RNA. Then, the amplified DNA was used for genotyping (Figure 1). A total of 140 µL of each serum was submitted for RNA extraction using a QIAmp Viral RNA Mini Kit (Qiagen, Germantown, MD, USA) following the manufacturer’s instructions. RNA samples were extracted in batches composed of a maximum of ten samples plus a negative extraction control (ultra-pure water). All the samples were tested for endogenous control (gene coding for β-actin, forward primer 5′ CCA ACC GCG AGA AGA TGA 3′ and reverse primer 5′ CCA GAG GCG TAC AGG GAT AG 3′) using a one-step real-time polymerase chain reaction (RT-qPCR) and a GoTaq® Probe 1-Step RT-qPCR System kit (Promega Corporation).

All the samples were tested using RT-qPCR, targeting the YFV 5′-UTR [24] (Figure 1). For the YFV molecular screening, negative (extraction control, a non-template control) and positive (YFV-17DD genomic RNA) controls were included. Briefly, 2.5 μL of RNA, 5 pmol of each primer were added to 5 μL of master mix (Promega Corporation) in a final volume of 10 μL. The amplification was performed on Applied Biosystems StepOne^TM^ Real-Time PCR Systems under the conditions: reverse transcription at 45 °C for 15 min, denaturation at 95 °C for 2 min and 40 cycles of 95 °C for 15 s and 60 °C for 60 s.

### 2.3. Yellow Fever Virus Genotyping

In order to optimize the genotyping tool, in silico phylogenetic analyses (Maximum-likelihood and Bayesian analysis, as described below) were first performed using partial NS5 nucleotide sequences from vaccine and all wild-type YFV genotypes (266 nucleotides, corresponding to positions 8993 to 9258 of YF-17D strain, GenBank accession number: X03700). This sequence corresponds to the target region of RT-qPCR previously demonstrated amplify sequences from different YFV genotypes, including vaccine virus [25].

Next, the RNA obtained from samples was used as a template in RT-qPCR targeting NS5 sequence (Figure 1). Samples from vaccinees were tested separately from the suspected cases of adverse vaccination events. In brief, 2.5 μL of RNA, 5 pmol of each primer (forward primer All_S 5′ TACAACATGATGGGGAARAGAGARAA 3′ and reverse primer All_AS2 5′ GTGTCCCAGCCNGCKGTGTCATCWGC 3′) were added to 5 μL of master mix (Promega Corporation) in a final volume of 10 μL. The amplification was performed on Applied Biosystems StepOne^TM^ Real-Time PCR Systems under the conditions: reverse transcription at 45 °C for 15 min, denaturation at 95 °C for min and 40 cycles of 95 °C for 15 s and 60 °C for 60 s. After that, a dissociation curve was carried out, with a gradual increase in temperature from 60 °C up to 95 °C. NS5 amplicons were purified and sequenced by the dideoxy method on an ABI3130 platform (Applied Biosystems). Raw data were analyzed, and final contigs were assembled using Geneious v. 9.1.8 (https://www.geneious.com).

The YFV nucleotide sequences obtained here (*n* = 46) were aligned with 111 vaccine and wild-type YFV sequences (GenBank accession numbers in Supplementary file S1), using Clustal W, implemented on MEGA7 [26]. The Kimura-2-parameters nucleotide substitution model with gamma distribution was selected [27] and used for the reconstruction of trees using the Maximum-likelihood method with 1000 bootstrap replicates, using MEGA7 [26]. Bayesian analyses were performed in parallel using BEAST package v.1.8.4 [28] with Markov Chain Monte Carlo algorithms. Input files for BEAST v1.8.4 were created with BEAUTi v.1.8.4 [28]. Runs were performed using Bayesian Skyline demographic coalescent models under the relaxed molecular clock. The best model was selected by comparing the marginal likelihood estimations, using path sampling (PS) method and stepping-stone sampling (SS) methods [29]. One hundred million chains were run, and after the convergence of parameters, verified with Tracer v.1.7.1 [30], the first 10 million steps were discarded. Uncertainties were addressed as the 95% Bayesian credible intervals (BCI). The trees were sampled at every 10,000 steps and then summarized in a maximum clade credibility tree using TreeAnotator v.1.8.4 [31]. Chains were run for three independent times, and data were combined using LogCombiner v.1.8.4. The final tree was visualized in FigTree v.1.4.3.

## 3. Results 

Phylogenetic in silico analyses of 111 NS5 partial sequences from vaccines and the wild-type viruses distinguished the vaccine viruses from all the wild-type YFV genotypes (data not shown). Thus, this approach was used for the investigation of adverse events following YF vaccination.

During the past YF outbreaks in Minas Gerais, our lab received sera from 21 patients with disease suspected to be related to adverse events following YF vaccination in 2017 and 2018. The patients reported the onset of the disease from day 1 to day 13 following vaccination, and they reported fever, headache, muscular pain, or vomiting, among other symptoms. All patients were clinically and laboratory diagnosed with YF by routine serologic or molecular tests performed by Reference laboratories linked to the State Health Secretary of Minas Gerais state.

Sera were used for RNA extraction, and samples were first screened for the presence of the endogenous control (β-actin). All samples were positive, with quantification cycle (Cqs) values between 21 and 29, indicating that the samples were suitable for RNA investigation. Although it has been demonstrated that β-actin mRNA can decrease over time following blood collection [32], the samples were adequately stored (liquid nitrogen or at −70 °C), and endogenous control was positive in all the samples.

Out of 21 investigated samples of suspected adverse events following YF vaccination, 19 were positive for the presence of YFV RNA using RT-qPCR targeting 5´UTR, however, only 17 exhibited NS5 amplicons (Table 1). All positive controls (sera from 25 healthy vaccinees and four patients with wild-type YFV infection) presented the expected 5´UTR and NS5 amplicons (Table 1).

**Figure 2:**
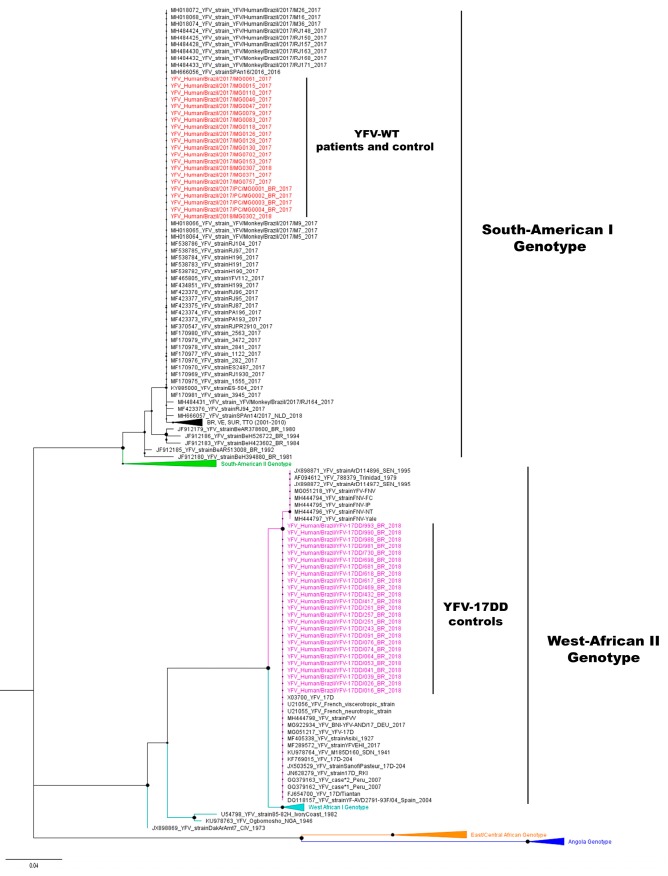
Maximum Likelihood tree of yellow fever virus. The maximum clade credibility tree inferred using 157 yellow fever virus (YFV) sequences (213 nt) is shown (corresponding to position 9020 to 9232 compared to the nucleotide sequence of YFV-17D, GenBank accession number: X03700). The bootstrap values (1.000 replicates) are represented by circles drawn in scale in the nodes. YFV wild-type and YFV-17DD sequences generated in this study are shown in red and pink, respectively. The clade containing samples from genotype South-American I is represented in black (some strains from Brazil, Venezuela, Suriname, and Trinidad and Tobago from 2011 to 2010 are collapsed). For clarity purposes, some branches representing different genotypes were collapsed and coloured as follows: South-American II (green), West African I (light blue), East/Central African (orange), and Angola (dark blue), respectively. The tree was reconstructed using the nucleotide substitution model kimura 2-parameters with 4-categories gamma distribution. The analysis was performed using MEGA7 and the tree visualised in FigTree v1.4.3.

NS5 amplicons were sequenced and phylogenetic trees were reconstructed using the 46 YFV nucleotide sequences obtained here (GenBank accession numbers: MN613537–MN613582) and 111 sequences obtained from Genbank (Supplementary file S1). Phylogenetic analyses based on Maximum likelihood (Figure 2) or Bayesian analysis (data not shown) indicated the clustering of South American I, South American II, West-African I, West-African II, and East/Central African genotypes in well-supported clades (Figure 2). YFV nucleotide sequences obtained from the 17 patients, suspected to have adverse event following YF vaccination, and from four naturally infected patients were identical to sequences of Brazilian wild-type YFV belonging to South American I genotype and clustered within these later strains (Figure 2). YFV nucleotide sequences obtained from 25 health primary vaccinees were identical to YFV-17DD sequences and grouped with vaccine strains, within the West-African II clade.

We received the results of anti-YFV IgM test (*in house* MAC-Elisa) of some patients performed at Fundação Ezequiel Dias (FUNED). Nine patients were anti-YFV IgM negative, and three were anti-YFV IgM positive (Table 1). It was possible to genotype YFV in samples collected from the second to the 16th day after the onset of symptoms, including three IgM-positive samples (Table 1).

## 4. Discussion

During the last YF outbreaks in Brazil, several suspected cases of adverse events following YF vaccination were reported [23]. We received samples from 21 suspected cases for viral genotyping, and we were able to genotype the virus in 17 cases. In later phases of infection, after seroconversion, viral load is usually low and could impair genome detection, nevertheless, we were still able to detect and genotype YFV in three samples in which anti-YFV IgM was detected. Samples from 17 patients were genotyped as wild-type YFV, as well as the four wild-type positive controls, while the vaccinees’ samples were classified as vaccine strains. The Bayesian analysis confirmed the results obtained by the Maximum-likelihood analysis, showing the suitability of this tool for YFV genotyping.

Here we successfully genotyped YFV samples belonging to South American I and West African II genotypes, containing the vaccine strains. One limitation of our study is the lack of YFV strains belonging to all genotypes to be experimentally tested by our tool, however, in silico analysis suggested that this protocol could also be used for other genotypes. Four cases remained inconclusive which could be due to (i) low viral genomic loads in samples, (ii) differences in sensitivity of PCR protocols with different targets (5′-UTR and NS5 regions), or (iii) due to mutations at primer annealing site at NS5 region. For those cases, other genotyping approaches could be used [16,17].

Fischer and colleagues [16] described an RT-qPCR capable of distinguishing a South American genotype from YF vaccine strains. A protocol based on nucleotide amplification, deep sequencing and phylogenetic analysis has also been proposed by Faria and colleagues [17]. Here, we used a protocol combining RT-qPCR, dideoxy sequencing and sequence analyses for the genotyping of YFV. RT-qPCR is simple, fast and responsive, with real-time results [33]. Sequencing of small fragments by dideoxy methodology [34] is cheap and needs less specialized labour demand for interpretation of the results when compared with third-generation sequencing techniques [35]. In that way, this tool could be suitable to different laboratories for YFV genotyping, even in some less equipped laboratories. Although genome detection and genotyping are essential to distinguish the adverse events from natural infection cases, all available approaches have limitations. In addition, no matter what protocol based on genome amplification is used, one should always keep in mind that viremia or RNAmia could be no longer detected in later days of infection. This fact reinforces the need for alternative methods based on different biomarkers to investigate adverse events following YF vaccination, in different phases of the YF course, supporting the control of the disease.

After an intense YF vaccination campaign in Minas Gerais state, 264 cases were classified as suspected adverse events [23]. Only one case (not investigated here) was confirmed to be related to YFV-17DD vaccination by State Health Secretary of Minas Gerais in 2018 [23]. Here, we contributed with the investigation of 21 suspected cases and only wild-type YFV was detected in 17 cases. Due to recent massive YF outbreaks in Brazil, the Ministry of Health expanded the vaccination recommendation area in the country. In that way, the genotyping protocol presented here could be beneficial to the investigation of future suspected adverse events following vaccination.

Although vaccination is the most powerful tool for yellow fever prevention and control, many challenges remain. The *Eliminating Yellow Fever Epidemics* (EYE) is a long-term global strategy aiming at eliminating yellow fever epidemics by 2026, based on achieving three main objectives. These aims include (i) to protect at-risk populations, (ii) to prevent international spread and (iii) to contain outbreaks rapidly. To attain this aim, it is necessary to raise population immunity levels and achieve sustained high yellow fever vaccination coverage in risk areas [36]. However, mass vaccination campaigns are usually associated with an increase in adverse events following vaccination [7,11]. The diagnosis of adverse events is made by temporal association with YF vaccination, clinical manifestation, and the detection of IgM or YFV RNA [6,9]. The testing solely based on IgM detection is not an assertive indicator of natural infection or adverse event following YF vaccination [37]. This particularly deserves caution when considering the occurrence of suspected cases of adverse events following YF vaccination (i) in YF risk areas, (ii) during YF outbreaks, (iii) in co-circulation of flaviviruses (given the flaviviruses antibody cross-reactivity), and (iv) during massive vaccination campaigns [7,9].

The straightforward flow of the genotyping tool presented here could improve the capacity to discriminate YF adverse event following vaccination from natural infections in YF endemic areas since it does involve a less infrastructure. Once this genotyping tool is implemented, this will allow viral genotyping in a timely manner during outbreaks or vaccination campaigns. This would enable the rapid resolution of cases and return to the population, increasing the vaccination adhesion and further vaccination coverage supporting YF control.

## 5. Conclusions

Here we described a new YFV genotyping tool, based on RT-qPCR and dideoxy sequencing. We used this tool to investigated adverse events following Yellow fever vaccination, after a mass vaccine campaign in Minas Gerais. We solved 17 suspected adverse events following YF vaccination cases and all the cases were classified as wild-type YFV genotype. The genotyping tool was efficient in distinguishing the vaccine from wild-type virus, and it has the potential to be used for the differentiation of all yellow fever virus genotypes.

## Figures and Tables

**Table 1:** Investigation of adverse events following yellow fever vaccination.

ID	Age	Gender	Day of YFV-17DD Vaccination	Day of Onset of Disease	Tested Sample					GT
					Days After		RT-qPCR (Target Region)		IgM	
					Symptoms	Vaccination	5′-UTR ^1^	NS5 ^2^		
1	57	M	23/1/17	23/1/17	3	3	Positive	Positive	N/A	WT
2	15	M	19/1/17	20/1/17	3	4	Positive	Positive	N/A	WT
3	38	F	13/1/17	13/1/17	7	7	Positive	Positive	Positive	WT
4	49	F	23/1/17	23/1/17	4	4	Positive	Positive	N/A	WT
5	51	M	14/1/18	16/1/18	2	4	Positive	Positive	Negative	WT
6	61	M	15/1/18	15/1/18	2	2	Positive	Positive	Negative	WT
7	22	M	23/1/17	23/1/17	5	5	Positive	Positive	N/A	WT
8	39	M	16/1/17	16/1/17	3	3	Positive	Positive	N/A	WT
9	42	M	16/1/17	17/1/17	3	4	Positive	Positive	N/A	WT
10	36	F	10/1/17	18/1/17	5	14	Positive	Positive	Positive	WT
11	37	F	13/1/17	23/1/17	5	16	Positive	Positive	Negative	WT
12	33	F	16/1/17	22/1/17	5	12	Positive	Positive	Negative	WT
13	34	M	20/1/17	21/117	6	8	Positive	Positive	Positive	WT
14	62	F	14/1/17	17/1/17	5	9	Positive	Positive	Negative	WT
15	57	M	20/1/17	21/1/17	8	9	Positive	Positive	N/A	WT
16	60	M	10/1/17	22/1/17	3	16	Positive	Positive	Negative	WT
17	42	M	15/1/17	17/1/17	6	9	Positive	Positive	Negative	WT
18	27	M	12/1/17	16/1/17	7	11	Positive	Negative	N/A	N/A
19	40	M	19/1/17	21/1/17	1	2	Positive	Negative	N/A	N/A
20*	48	F	14/1/17	18/1/17	7	12	Negative	Negative	Negative	N/A
21**	32	F	14/1/17	25/1/17	4	16	Negative	Negative	Negative	N/A
22	47	M	16/2/18	N/A	N/A	5	Positive	Positive	N/A	17DD
23	54	F	16/2/18	N/A	N/A	6	Positive	Positive	N/A	17DD
24	45	F	23/2/18	N/A	N/A	6	Positive	Positive	N/A	17DD
25	48	M	23/2/18	N/A	N/A	4	Positive	Positive	N/A	17DD
26	26	F	21/2/18	N/A	N/A	5	Positive	Positive	N/A	17DD
27	42	F	28/2/18	N/A	N/A	5	Positive	Positive	N/A	17DD
28	45	F	22/2/18	N/A	N/A	5	Positive	Positive	N/A	17DD
29	75	M	23/2/18	N/A	N/A	5	Positive	Positive	N/A	17DD
30	42	M	23/3/18	N/A	N/A	6	Positive	Positive	N/A	17DD
31	51	M	26/2/18	N/A	N/A	8	Positive	Positive	N/A	17DD
32	16	F	21/2/18	N/A	N/A	5	Positive	Positive	N/A	17DD
33	58	F	21/2/18	N/A	N/A	5	Positive	Positive	N/A	17DD
34	7	M	21/2/18	N/A	N/A	5	Positive	Positive	N/A	17DD
35	65	M	15/2/18	N/A	N/A	4	Positive	Positive	N/A	17DD
36	24	M	16/2/18	N/A	N/A	4	Positive	Positive	N/A	17DD
37	50	M	19/2/18	N/A	N/A	4	Positive	Positive	N/A	17DD
38	54	M	1/3/18	N/A	N/A	5	Positive	Positive	N/A	17DD
39	39	F	1/3/18	N/A	N/A	6	Positive	Positive	N/A	17DD
40	59	F	5/3/18	N/A	N/A	4	Positive	Positive	N/A	17DD
41	55	M	9/3/18	N/A	N/A	5	Positive	Positive	N/A	17DD
42	60	M	19/3/18	N/A	N/A	1	Positive	Positive	N/A	17DD
43	62	M	19/2/18	N/A	N/A	4	Positive	Positive	N/A	17DD
44	48	M	23/2/18	N/A	N/A	5	Positive	Positive	N/A	17DD
45	55	M	21/2/18	N/A	N/A	5	Positive	Positive	N/A	17DD
46	28	M	22/2/18	N/A	N/A	5	Positive	Positive	N/A	17DD
47	31	M	N/A	6/1/18	4	N/A	Positive	Positive	N/A	WT
48	41	M	N/A	10/1/18	7	N/A	Positive	Positive	N/A	WT
49	65	M	N/A	13/1/18	4	N/A	Positive	Positive	N/A	WT
50	62	M	N/A	15/1/18	3	N/A	Positive	Positive	N/A	WT

ID: patient identification. GT: genotyping. M: male. F: Female. YFV: yellow fever virus. WT: wild-type. N/A: not available. ^1^ Domingo et al (2012). ^2^ Patel et al. (2013). * the YFV infection was further confirmed by MAC-ELISA in a sample collected on 28 January 2017 and ** the YFV infection was further confirmed by RT-qPCR in a sample collected on 27 January 2017 by Reference Laboratory linked to the State Health Secretary of Minas Gerais state. Suspected cases of adverse events following YF vaccination investigated here (patients 1 to 21) are highlighted in grey. Healthy YFV-17DD primary vaccines are numbered from 22 to 46, and wild-type YFV naturally infected patients are numbered from 47 to 50.

## Supplementary Materials

File S1: GenBank accession numbers and alignment used for phylogenetic analyses.
